# Transcriptome profiling of microRNAs associated with latent autoimmune diabetes in adults (LADA)

**DOI:** 10.1038/s41598-019-47726-z

**Published:** 2019-08-05

**Authors:** Ke Yu, Zhou Huang, Jing Zhou, Jianan Lang, Yan Wang, Xingqi Yin, Yuan Zhou, Dong Zhao

**Affiliations:** 1 Beijing Key Laboratory of Diabetes Prevention and Research, Department of Endocrinology, Lu He Hospital, Capital Medical University, Beijing, 101149 China; 2 Department of Biomedical Informatics, School of Basic Medical Sciences, Center for Noncoding RNA Medicine, Peking University, Beijing, 100191 China

**Keywords:** Metabolic disorders, Endocrine system and metabolic diseases

## Abstract

LADA (latent autoimmune diabetes in adults), a special subtype of type 1 diabetes, turns out to exhibit phenotypes mimicking the type 2 diabetes, which results in serious misdiagnosis issues. In order to better distinguish LADA from other diabetes subtypes, specific diagnostic and prognostic biomarkers of LADA are required. Circulating microRNAs (miRNAs) are recently shown to be promising biomarkers for disease diagnosis and subtyping. In this study, serum samples from LADA patients and type 2 diabetes patients were collected during the first diagnosis of diabetes and the miRNA transcriptomes of these patients and healthy individuals were profiled. Comparative analysis shows that the differentially expressed miRNAs between groups and their predicted target genes are enriched for several functions including immune regulation. Besides, a few miRNAs showing distinct expression pattern in LADA patients could discriminate LADA from type 2 diabetes, as validated by further qRT-PCR assay. In all, our study implies potential miRNA biomarkers which would be investigated in further clinical researches.

## Introduction

The morbidity of diabetes has increased rapidly worldwide, which places heavy economic and social burden. Diabetes is mainly classified into two types, i.e. type 1 diabetes (T1D) and type 2 diabetes (T2D), according to the etiology of diseases. For most cases, these two types are clearly distinguishable as per the early symptoms. Type 1 diabetes is often characterized by rapid autoimmune damage of pancreas islet β cells, which further results in insulin treatment dependency of the patient. However, latent autoimmune diabetes in adults (LADA), one special subtype of type 1 diabetes, is featured by slow damage of islet β cells^1^. Therefore, LADA patients often show early signs mimicking type 2 diabetes, which results in nonnegligible misdiagnosis rate. Indeed, it has been estimated the incidence of LADA is about 6% among newly diagnosed type 2 diabetes patients^2^. Clinical trials have suggested that insulin therapy, the golden-standard treatment of conventional type 1 diabetes, also clearly applicable to the LADA patients^3^. Besides, immunosuppressant and islet β cell-specific antigen, which counteract the autoimmune process against islet β cell, could also improve the function of islet β cell of LADA patients, especially during the early phase of the disease^4,5^. Nevertheless, without accurate diagnosis of LADA, timely insulin therapy is not possible.

Currently, the diagnostic criteria of LADA are still under debates. Because the slow progression of the disease, the onset of adult disease would be one indication of LADA. However, according to the statistics from LADA China Study, the age of onset could vary significantly, and therefore the onset of adult disease is not a confident exclusion standard of LADA^6^. Besides, the islet autoantibody level, an indicator of autoimmune damage of islet β cells, could also be a diagnosis biomarker of LADA. The glutamic decarboxylase antibody (GADA) titer could differ between acute and slowly progressive type 1 diabetes, and indeed a subgroup of LADA (known as LADA subtype 1), could be characterized by high titer of GADA^1^. However, for other LADA patients (known as LADA subtype 2), the GADA level is not prominently elevated but turns out to be similar to that of type 2 diabetes^7^. Therefore, novel biomarkers, including novel autoimmune biomarkers and other types of molecular biomarkers, for LADA diagnosis are continuously demanded^8^.

MicroRNAs are a class of non-coding single-stranded small molecule RNA with the length of 19~22 nucleotides, which can regulate the expression of hundreds of target genes^9^. As a key post-transcriptional regulatory factor, miRNAs can not only affect growth and development, but also influence the occurrence and progression of cancers and other physiological and pathological processes^10^. MicroRNAs (miRNAs) were recently found to be stably presented in blood and other body fluids including urine, saliva, amniotic fluid and breast milk, and such circulating microRNAs could therefore serve as the promising biomarkers for disease diagnosis^11–13^. For example, according to the circulating miRNA profiles from 265 patients with different degrees of metabolic syndrome, it was found that the expression of miR-27a and miR-320a were significantly correlated with the increase of the fasting blood glucose, suggesting that miRNAs may be potential biomarkers of type 2 diabetes^14^. Likewise, a cohort study conducted on patients with initial diagnosis of type 1 diabetes^15^ was found that a group of miRNAs (miR-24, miR-25, miR-27a, miR-27b, miR-29a, miR-30a-5p, miR-152, miR-181a, miR-200a and miR-210) were significantly different from the control group. Among these miRNAs, miR-25 was found to be related to the function of remaining islet β cells and good blood glucose control in 3 months after the onset of the disease. Besides, some miRNAs can regulate immune cell function (miR-31, miR-146a, miR-155, miR-181a and miR-199a) or islet β cell function (miR-9 and miR-34a)^16^, and therefore could also be potential biomarkers for type 1 diabetes diagnosis. Finally, in animal models, miR-375 is highly expressed in the islet, and changes in miR-375 level subsequently causes the death of islet β cell within 1 week duration, suggesting miR-375 could also be used as a biomarker to predict the progression of type 1 diabetes^17^.

In all, circulating microRNAs have become an attractive new class of biomarkers to predict occurrence and development of diabetes, the risk and development of chronic complications of diabetes, and evaluate the efficacy of intervention. Nevertheless, the investigation about the circulating miRNAs signatures in LADA patients is still limited. Recently, Seyhan *et al*. have performed a pilot cross-sectional study to compare the miRNA profiles between different (sub) types of diabetes, including LADA^18^. Unfortunately, however, although few miRNAs like miR-34a and miR-29a show trends of de-regulation in LADA, no miRNAs meet the statistical significance threshold, and therefore the predictor based on miRNA profiles fails to distinguish the LADA patients from other diabetes patients. One confounding factor should be the anti-diabetes therapies like metformin, sulfonylureas, or DPP-4 inhibitor, which may alter the circulating miRNA expression pattern of the diabetes patients^18^. Therefore, in this study, we intentionally collected blood samples from the patients during the first diagnosis of diabetes. The circulating miRNAs in patients’ blood samples were profiled by using microarray technique. The functions of de-regulated miRNAs in LADA were analyzed by bioinformatics tools, and several consistently de-regulated miRNAs were suggested as the potential biomarker for the early diagnosis of LADA.

## Results

### MicroRNA expression profiles

**Figure 1:**
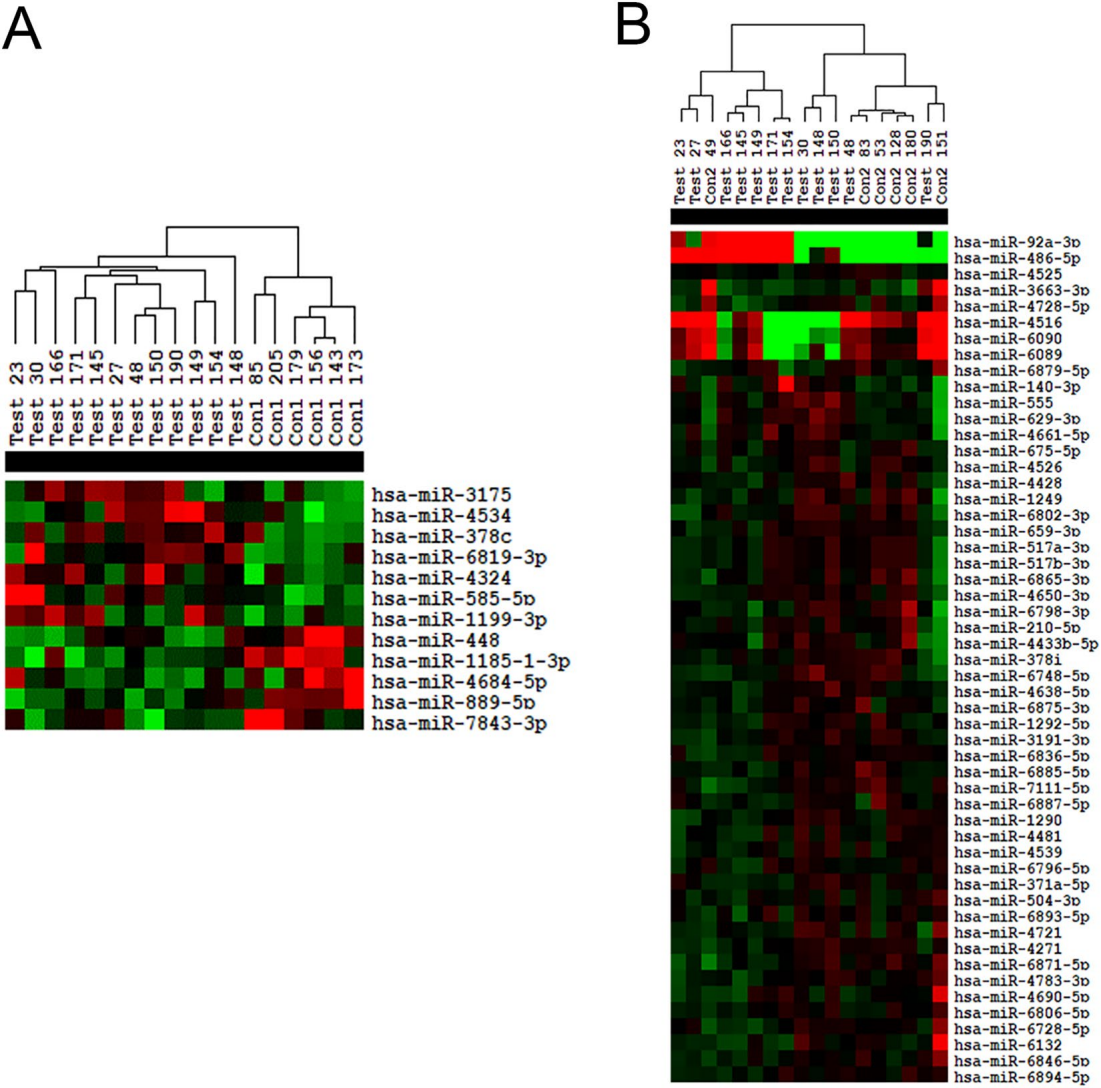
The heatmaps of differentially expressed microRNAs in two comparisons. (**A**) Test_vs_Con1 comparison. (**B**) Test_vs_Con2 comparison. The differentially expressed miRNAs were screened with the threshold of fold change >1.2 and P-value < 0.05.

We isolated serums from the blood samples of three groups of people (Table 1), which were used for subsequent microarray analysis (Test group: 12 LADA patients; Con1 group: 6 healthy people; Con2 group: 6 patients of type 2 diabetes). To find the crucial difference in gene expression between the Test group and other two control groups, we screened differentially expressed miRNAs with the threshold of fold change >1.2 and P-value < 0.05. Finally, we identified 12 miRNAs in Test_vs_Con1 comparison and 53 in Test_vs_Con2 comparison. The heat maps of the differentially expressed miRNAs in two comparisons are shown in Fig. 1A,B, respectively. Among the Test group or the Con1 group, the miRNA expression profiles have consistent changes, but the expression profiles in the Con2 group are varied from person to person. Nevertheless, it could be observed that the serum miRNA expression profiles from the same disease tend to be clustered together. Besides, we also intuitively confirmed the differentially expressed miRNAs by the scatter plots and volcano plots of the miRNAs, as shown in Supplementary Fig. S1. The detailed list of differentially expressed miRNAs is in Supplementary Table S1.

**Table 1:** Clinical characteristics of the study groups.

	Normal	Type 2 diabetes	LADA	P^a^	P^b^
Patient, n	6	6	12	—	—
Women, n (%)	2 (33.3%)	2 (33.3%)	4 (33.3%)	—	—
Age, years	51.50 (36.75,57.75)	45.50 (31.00,59.25)	51.00 (39.00,59.00)	0.960	0.688
BMI, kg/m2	24.43 (20.09,27.07)	26.85 (24.86,28.10)	21.52 (19.04,29.03)	0.833	0.282
HbA1c, %	5.450 (5.375,5.525)	9.500 (8.275,10.20)	7.700 (6.800,9.800)	0.001^a^	0.451
Fasting insulin, mU/L	10.51 (8.573,18.53)	13.29 (11.30,23.99)	15.15 (8.490,18.30)	0.651	0.725
Fasting C-peptide, ng/ml	2.545 (2.115,3.040)	2.840 (1.373,3.878)	2.740 (1.630,4.280)	0.880	0.960
Fasting blood glucose, mmol/L	5.295 (5.260,5.915)	11.72 (9.375,12.65)	10.29 (7.675,13.27)	0.006 ^a^	0.743
ZnT8-Ab positive, n (%)	0	0	10 (83.3%)	—	—
GAD-Ab positive, n (%)	0	0	6 (50.0%)	—	—
Triglyceride, mmol/L	1.230 (1.010,2.068)	1.875 (1.138,2.505)	1.460 (1.023,2.045)	0.607	0.426
Total cholesterol, mmol/L	4.780 (3.983,6.060)	4.485 (4.308,5.763)	4.620 (4.015,5.573)	0.673	0.607
HDL-C, mmol/L	1.275 (1.170,1.555)	1.080 (0.9950,1.150)	1.050 (0.9525,1.413)	0.223	1.00
LDL-C, mmol/L	2.860 (2.483,4.175)	2.985 (2.465,3.640)	2.985 (2.465,3.640)	0.963	0.743
C- reactive protein, mg/L	1.295 (0.8200,1.875)	2.665 (1.028,17.41)	1.750 (1.085,3.685)	0.303	0.733

^a^p value for comparisons between Normal and LADA; ^b^p value for comparison between type 2 diabetes and LADA.

### Functional enrichment analysis of differentially expressed microRNAs

**Figure 2:**
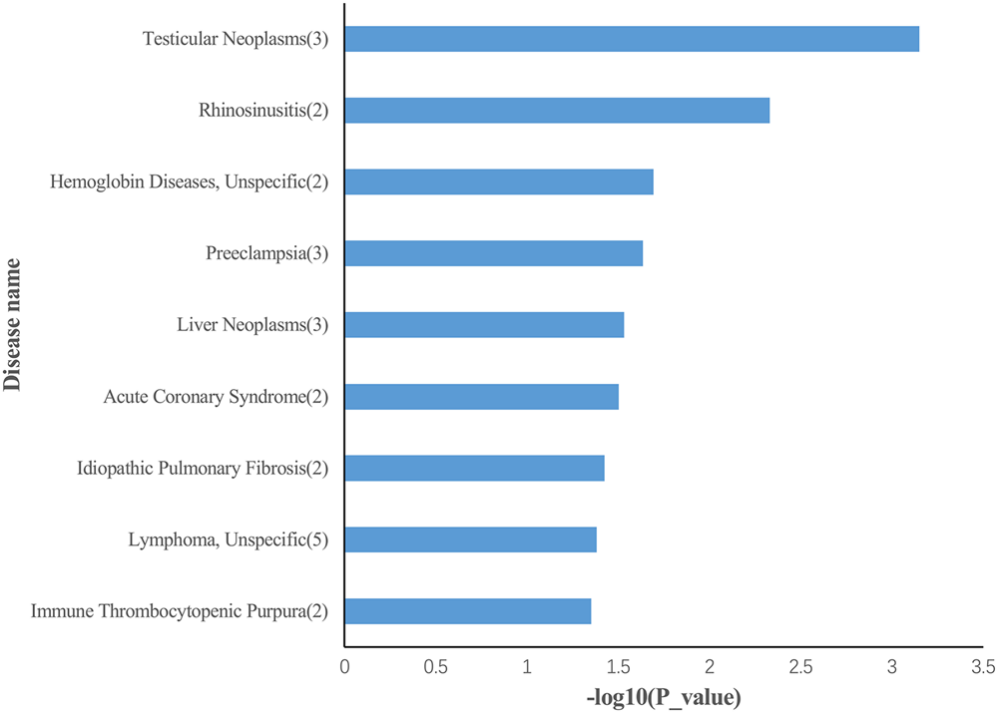
TAM 2.0 miRNA-disease association analysis results of differentially expressed miRNAs in Test_vs_Con2 comparison.

To explore the associated human diseases and enriched functions of the 65 differentially expressed miRNAs, we used TAM 2.0 tool^19^ to perform functional enrichment analysis. Unexpectedly, the Test_vs_Con1 differentially expressed microRNAs are associated with the habitual abortion disease (P-value = 0.0183). This is likely resulted from the incomplete miRNA-disease annotation of the Test_vs_Con1 differentially expressed miRNAs. Many of them are miRNAs without sufficient functional investigations, as indicated by their late miRNA series numbers. Indeed, the better annotated Test_vs_Con2 differentially expressed microRNAs exhibit several interesting disease associations, as shown in Fig. 2. Several immune-related terms, including rhinosinusitis (P-value = 4.68E-03) and immune thrombocytopenic purpura (P-value = 0.0445) are enriched, which signifies the etiological discrimination between LADA and type 2 diabetes.

### MicroRNA target gene prediction and functional enrichment analysis of target genes

**Figure 3:**
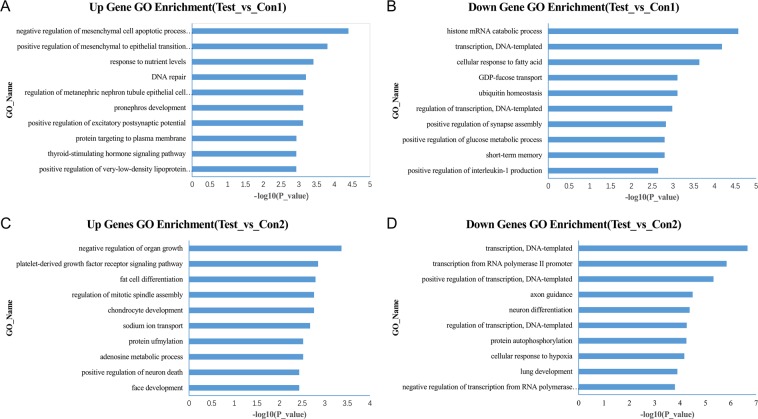
GO enrichment analysis of the miRNA target genes. (**A**) The enriched terms for the target genes of the up-regulated miRNAs in Test_vs_Con1 comparison. Only the top 10 enriched terms are shown. (**B**) The enriched terms for the target genes of the down-regulated miRNAs in Test_vs_Con1 comparison. (**C**) The enriched terms for the target genes of the up-regulated miRNAs in Test_vs_Con2 comparison. Only the top 10 enriched terms are shown. (**D**) The enriched terms for the target genes of the down-regulated miRNAs in Test_vs_Con2 comparison.

The functional enrichment analysis of miRNAs is limited by the current miRNA functional annotations and the results are not fully satisfactory. Therefore, we further performed target gene analysis of these differentially expressed miRNAs. First, target gene prediction were done by using the TargetScan^20^ and miRanda^21^ tools. Then we took the intersection of the two prediction results to obtain high confidence miRNA targets. Finally, we obtained 1469 target genes for the Test_vs_Con1 comparison and 11782 genes for the Test_vs_Con2 comparison. We used these gene lists as the input to the functional enrichment analysis tool clusterProfiler^22^. We mainly focused on the biological process terms of the Gene Ontology. The top ten enriched terms for each comparison were shown in Fig. 3. From the results, it can be found that these genes are widely in association with the metabolic and gene expression-related functions. Interestingly, target genes of down-regulated miRNAs in Test_vs_Con1 comparison is related to the positive regulation of glucose metabolic process. By summarizing four enrichment analysis results, we noted that these target genes are also enriched in DNA-templated transcription (Test_vs_Con1, down, P-value = 6.63E-05; Test_vs_Con2, down, P-value = 2.18E-07).

### Pathway enrichment analysis of the target genes

**Figure 4:**
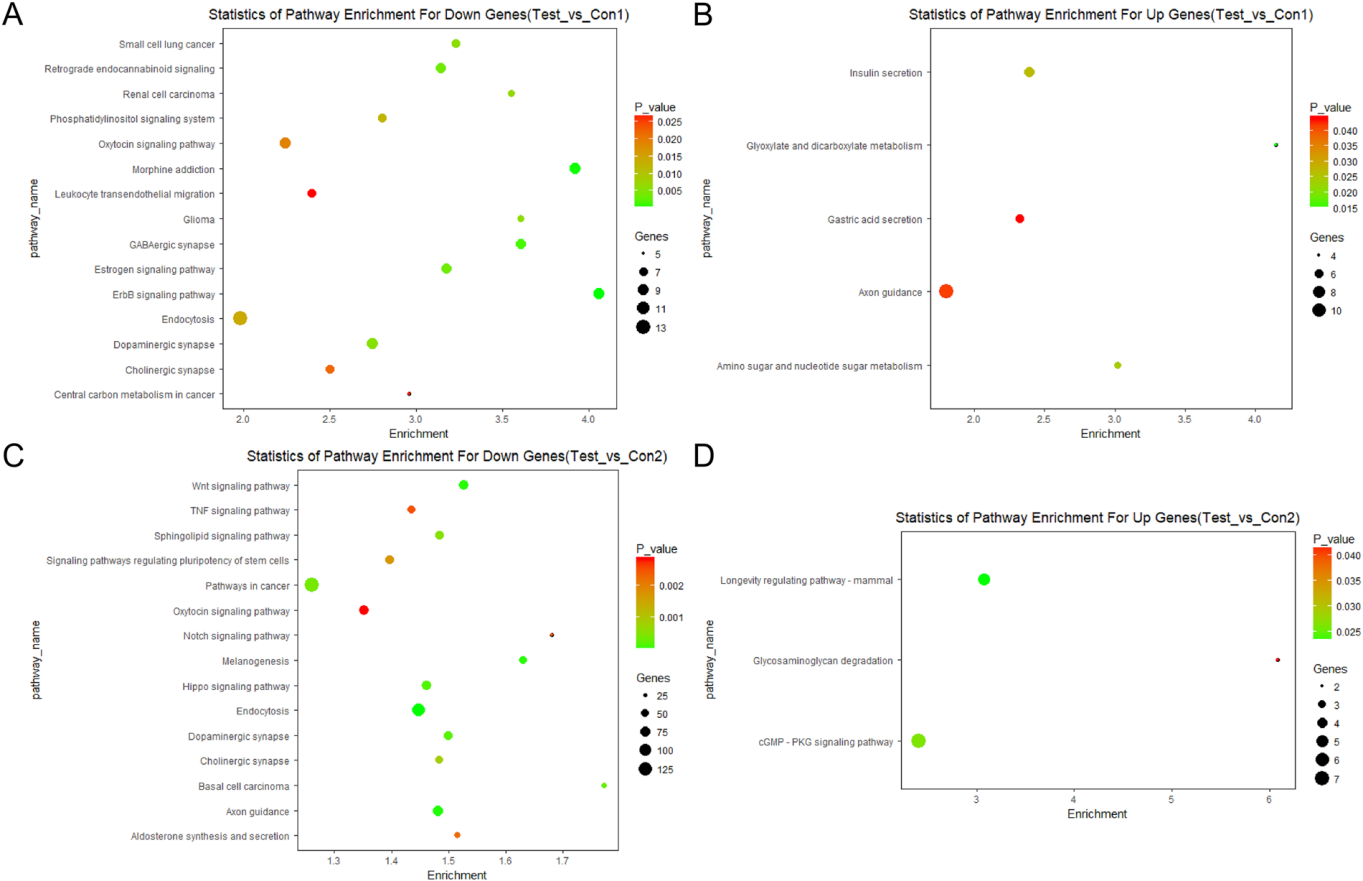
Bubble plots depicting KEGG pathway analysis results of the target genes. The color depth represents P-value and the size of the dots represents the number of genes in each pathway. (**A**) The enriched terms for the target genes of down-regulated miRNAs in Test_vs_Con1 comparison. Only the top 15 enriched terms are shown. (**B**) The enriched terms for the target genes of up-regulated miRNAs in Test_vs_Con1 comparison. (**C**) The enriched terms for the target genes of down-regulated miRNAs in Test_vs_Con2 comparison. Only the top 15 terms are shown. (**D**) The enriched terms for the target genes of up-regulated miRNAs in Test_vs_Con2.

The top significant KEGG pathway terms (P-value < 0.05) are further listed in Fig. 4. From these bubble plots, several metabolic pathways including glyoxylate and dicarboxylate metabolism (P-value = 0.0147), amino sugar and nucleotide sugar metabolism (P-value = 0.0240) and insulin secretion (P-value = 0.0268), are all related to glucose metabolism. Notably, two pathways related to the etiology of LADA and type 2 diabetes are also highlighted. First, it has been reported that the stimulated insulin secretion capacity of LADA was intermediate between the ability of the types 1 and 2 diabetes^23^ and our analysis result indicates that the insulin secretion (P-value = 0.0268) is also one of the top enriched pathways. Second, we find the enriched term of thyroid hormone signaling pathway (P-value = 0.0291), which could also have a relation with LADA. The biomarker of thyroid disease turns out to be associated with the autoimmune phenotype in type 2 diabetic patients^17^. And the top enriched cGMP-PKG signaling pathway (P-value = 0.0262), one pathway closely related to thyroid disease, has been proved to regulate insulin secretion^24^. Therefore, the association with thyroid hormone signaling pathway is in accordance with the autoimmune nature of LADA.

### Potential miRNA biomarkers discriminating LADA

**Figure 5:**
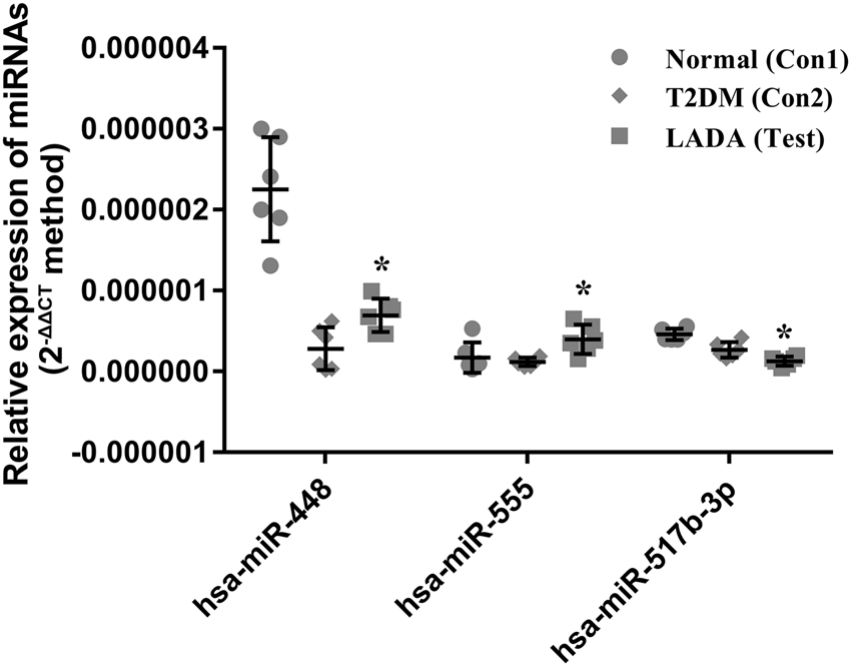
The qRT-PCR validation of the differential expression of the potential miRNA biomarkers. The candidates for qRT-PCR validation was selected from the consistently changed miRNAs in LADA from Table 2. The serum expression level of hsa-miR-448, hsa-miR-555 and hsa-miR-517a-3p in LADA (Test), healthy (Con1) and type 2 diabetes (Con2) groups were compared and depicted by dot box plots. The three horizontal bars in the box indicate mean ± SD, respectively. The expression of each sample is shown as the jitter dots on the plots. * indicates p < 0.05. N = 6 per group, i.e. in total 18 blood samples were tested by qRT-PCR.

Because the differentially expressed miRNAs in Test_vs_Con1 and Test_vs_Con2 are rarely shared (Supplementary Table S1), we further screen miRNAs showing consistent changes in two comparisons. More specifically, we required a minimum fold change of 1.5 in one comparison and 2.25 in another, without any P-value requirement. As the result, two consistently up-regulated miRNAs (hsa-miR-93-5p and hsa-miR-555) and eight consistently down-regulated miRNAs were obtained (Table 2). These miRNAs could serve as potential biomarkers discriminating LADA patients from the healthy people and type 2 diabetes individuals. We analyze the significant disease associations of these miRNAs by using TAM 2.0. Indeed, these miRNAs are associated with metabolism and cancers like squamous cell carcinoma, laryngeal or hypopharyngeal (P-value = 0.0258) and liver neoplasms (P-value = 0.0459), which also implies their functional roles in LADA. Finally, three miRNAs (hsa-miR-448, hsa-miR-555 and hsa-miR-517b-3p) were selected for further qRT-PCR validation in an independent batch of samples. These candidates for qRT-PCR validation were selected from the consistently changed miRNAs in LADA (Table 2), which require the consistent miRNA de-regulation comparing to both healthy people and type 2 diabetes individuals. Such consistently changed miRNAs may be helpful to distinguish LADA patients from both healthy people and type 2 diabetes individuals. The qRT-PCR results are shown in Fig. 5, and the raw CT values are also available in Supplementary Table S2. The expression patterns of hsa-miR-555 and hsa-miR-517b-3p in LADA patients is in coincidence with the trends observed in miRNA transcriptomic profiling, where the LADA group exhibits the highest and the lowest expression levels, respectively. The expression level of hsa-miR-448 of LADA group turns out be in between the type 2 diabetes and healthy groups. Nevertheless, it shows significant differentially expression comparing to either group, indicating it could also be used to discriminate LADA patients. We also checked whether the microarray expression of these miRNAs among diabetes individuals correlates with the GAD antibody titer, one of the most accepted LADA molecular signature^1^. As shown in the Supplementary Fig. S2, hsa-miR-517b-3p expression shows significant negative correlation with GAD antibody titer (Pearson correlation = −0.684, P = 0.014), in line with its observed down-regulation in LADA patients in both microarray (Table 2) and qRT-PCR (Fig. 5) assay. Besides, the expression of hsa-miR-448 and hsa-miR-555 shows a trend of positive correlation with GAD antibody titer, but these correlations are not statistically significant, likely due to lack of enough LADA samples. In all, the qRT-PCR results further suggest the capability of circulating miRNAs, at least hsa-miR-517b-3p, as the LADA biomarker.

**Table 2:** Consistently changed miRNAs in LADA.

Group	miRNA_ID	Regulation
Con2 < Con1 < Test	hsa-miR-555	up
Con1 < Con1 < Test	hsa-miR-93-5p	up
Test < Con1 < Con2	hsa-miR-507	down
	hsa-miR-517a-3p	down
	hsa-miR-517b-3p	down
	hsa-miR-4691-3p	down
Test < Con2 < Con1	hsa-miR-370-5p	down
	hsa-miR-448	down
	hsa-miR-1236-3p	down
	hsa-miR-1267	down

## Discussion

At present, the understanding of LADA is still limited, and the diagnosis and treatment of LADA remain difficult and controversial. The recognized diagnostic criteria for LADA include adult onset, islet autoantibodies and insulin-free treatment for at least 6 months after diagnosis. Although the detection of autoimmune markers such as serum C peptide and GADA may contribute to differential diagnosis, it is difficult to make an early, definitive diagnosis of LADA, which caused the delayed or adverse treatment on the patients. LADA China investigators performed islet autoantibodies in 2,388 non-insulin-treated, newly diagnosed diabetic adult patients. 206 patients were found to be positive for islet autoantibodies, among which 138 patients with GADA (with a detection rate of 5.78%), only accounting for 67% of autoimmune diseases^25^. In clinical practice, we also found suspicious positive islet autoantibodies in LADA patients, and some of them have similar incipient symptoms as patients with type 2 diabetes. Unfortunately, they were given long-term treatment with oral sulfonylureas hypoglycemic drugs, which turned out to accelerate the progression of LADA. Therefore, early diagnosis and intervention LADA have a great significance for preserving the remaining islet β cell function, and delaying the onset of chronic complications and development of LADA.

Improving LADA diagnosis rate is the most basic step in the treatment of patients with LADA. We here investigated the expression profile of miRNAs in intravenous plasma of LADA patients and found the circulating microRNAs that were specifically de-regulated in plasma of LADA patients. It not only provides important theoretical basis and experimental data for the early differential diagnosis and treatment of LADA, but also opens a new direction for the discussion of the prevention and treatment of LADA. It also can be applied to the clinical treatment as a target molecule of drugs, providing a new direction for the early intervention treatment of LADA.

## Materials and Methods

### Ethics and consent declaration

The study protocol, which conforms to the provisions of the Declaration of Helsinki (as revised in Seoul, 2008), was reviewed and approved by the Hospital’s Ethics Committee of Beijing Luhe Hospital and the Ethics Committee of Capital Medical University. All the subjects of this study have signed informed consent.

### The clinical research objects

The subjects of this study are adult patients with primary diabetes who visited the endocrinology department of our hospital and healthy examined adults in physical examination center from January 2016 to December 2016. First, we divided the adult patients based on their serum islet autoantibodies including GAD, IA2, ZnT8, which were tested according to the previous protocol^3^. The antibody testing was performed in Second Xiangya Hospital. There are 12 cases of patients in the LADA group and 6 cases of patients in the type 2 diabetes group. Normal control group includes 6 cases of healthy examined adults at the same period. According to the power analysis, if we assume 1.5 fold change of expression level, with significance level α = 0.05, and the power β = 0.8, we could find 6 subjects per group is sufficient when the standard deviation of the expression level does not exceed 25% of the mean. This perquisite, as exemplified by the qRT-PCR validation results (Fig. 5), can at least be fulfilled by the top-ranked biomarker candidates.

### Patient inclusion and exclusion criteria

Inclusion criteria for patients with newly diagnosed diabetes: signed informed consent; age ≥18 years; screening and diagnosis of diabetes according to WHO (1999) diagnostic criteria for diabetes: fasting plasma glucose level ≥126 mg/dl (≥7.0 mmol/l), 2-h OGTT value ≥200 mg/dl (≥11.1 mmol/l) or random blood glucose level ≥200 mg/dl (≥11.1 mmol/l) with typical diabetic symptoms (polidipsia, polyuria, polyphagia and weight loss)

Exclusion criteria: younger than 18 years old; severe cardiovascular and cerebrovascular diseases; serious lung disease; a history of serious gastrointestinal diseases; a history of chronic liver disease or serum ALT or AST is 2.5 times higher than normal; chronic renal failure; previous diagnosis of type 1 diabetes, type 2 diabetes, gestational diabetes or other special type of diabetes is definitely; type 1 diabetes was clearly diagnosed at the initial visit.

### Plasma sample preparation and total RNA extraction

Plasma samples were prepared with EDTA anticoagulant tube. For each patient, 2 ml fasting venous blood was collected and centrifuged in 1 hour at room temperature (820 g, 4, Centrifugal 10 minutes). Approximately 1 ml of supernatant were drained into a new non-enzymatic centrifuge tube, centrifuged again (16000 g, 4, 10 min) to obtain the serum sample. The serum samples were reserved in −80 °C refrigerator. The total RNA was extracted in RNase-free condition (Beijing Compass Biotechnology). Nano Drop 1000 Spectrophotometer was used to determine the total RNA concentrations. RNA quality monitoring was performed by 2100 Bioanalyzer through RNA 6000 Pico LabChip Kit (Agilent, USA).

### Microarray analysis

We analyzed the plasma miRNAs expression profile of LADA patients with Affymetrix miRNA GeneChip miRNA 4.1 Array, which was completed by the Compass Biotechnology, China. This Affymetrix miRNA Array provides 100% miRBase v20 coverage of 5214 human, mouse and rat miRNAs, together with 3770 probe sets unique to human, mouse and rat pre-miRNA hairpin sequences. Tapman MicroRNA assays Kit (Ambion, USA) was applied for labeled, condensed and hybridized of miRNAs. The chip image was scanned by Genepix 4000B scanner and the data was analyzed by Genepix Pro 6.0. The data was processed by overall normalization and logarithmic transformation, and clustering analysis was performed with Eisen CLUSTER and TREEVIEW software.

### MicroRNA target prediction and functional enrichment analysis

MiRNA target gene prediction were performed by TargetScan^20^ and miRanda^21^. For TargetScan, we used the software downloaded from its official website (http://www.targetscan.org/vert_72/) and analyzed with the Perl script. For miRanda, we ran the downloadable tool (http://www.microrna.org/) in a Java environment. At last, we took the intersection of the two prediction results to obtain high confidence miRNA targets.

TAM 2.0^19^ was used for miRNA functional enrichment analysis (http://www.lirmed.com/tam2/, queried in April, 2018). We chose the overrepresentation analysis function of TAM 2.0 and enabled the analysis of up- and down-miRNA sets in diseases. As for the functional enrichment of targets, we implemented the analysis pipeline in clusterProfiler^22^ package in R. To detect the overrepresentation of Gene Ontology terms among the large list of target genes, the Parent-Child-Intersection method was used for enrichment analysis and Benjamini-Hochberg was used for multiple test correction. Note that only manually curated associations (i.e. all evidence codes except IEA, ND and NR) were considered for the enrichment analysis. As for the pathway enrichment analysis, after parsing the whole KEGG database, all target genes involved in specific pathways were extracted, and the hypergeometric test was used to calculate the pathway enrichment, the FDR method was used to adjust the P-values for multiple comparisons.

### MicroRNA extraction and quantitative real time polymerase chain reaction (qRT-PCR)

The potential miRNA biomarkers were tested by qRT-PCT by using an independent batch of samples, including 6 subjects for each group (Test, Con1 and Con2). All the subjects have signed informed consent. Total miRNA was extracted from the serum samples by a miRcute Serum/Plasma miRNA Isolation Kit (TIANGEN, Beijing, China) according to the manual. Single-stranded cDNA was obtained from the extracted miRNA, and qRT-PCR was performed to verify the expression of hsa-miR-448, hsa-miR-555 and hsa-miR-517b-3p through a miDETECT Tract^TM^ miRNA qRT-PCR Starter Kit (RiboBio, Guangzhou, China). The following primers were obtained from RiboBio Company (Guangzhou, China), hsa-miR-448: UUGCAUAUGUAGGAUGUCCCAU; hsa-miR-555: AGGGUAAGCUGAACCUCUGAU; hsa-miR-517b-3p: AUCGUGCAUCCCUUUAGAGUGU. Finally, the miRNA relative expression levels in serum were derived by normalizing to the level of the control cel-miR-39-3p using the 2^−ΔΔCT^ method.

## Supplementary information

Supplementary Figures S1-S2 and Supplementary Tables S1-S2
