# Supplementary material for: Transcriptome profiling of microRNAs associated with latent autoimmune diabetes in adults (LADA)
Source: Sci Rep. 2019 Aug 5;9:11347. doi: 10.1038/s41598-019-47726-z (PMC6683294; doi:10.1038/s41598-019-47726-z)
Supplement: Supplementary file 1 — Supplementary Figures S1-S2 and Supplementary Tables S1-S2 [file 41598_2019_47726_MOESM1_ESM.docx]

**Transcriptome profiling of microRNAs associated with** **latent autoimmune diabetes in adults (LADA)**

Ke Yu^1,+^, Zhou Huang^2,+^, Jing Zhou^1^, Jianan Lang^1^, Yan Wang^1^, Xingqi Yi^1^, Yuan Zhou^2,*^, Dong Zhao^1,*^

^1^ Beijing Key Laboratory of Diabetes Prevention and Research, Department of Endocrinology, Lu He Hospital, Capital Medical University, Beijing 101149, China

^2^ Department of Biomedical Informatics, School of Basic Medical Sciences, Center for Noncoding RNA Medicine, Peking University, Beijing 100191, China

^+^These authors contributed equally to this work.

* To whom the correspondence should be addressed:

E-mail: Dong Zhao ([zdoc66@126.com](mailto:zdoc66@126.com)) and Yuan Zhou ([zhouyuanbioinfo@hsc.pku.edu.cn](mailto:zhouyuanbioinfo@hsc.pku.edu.cn))

**
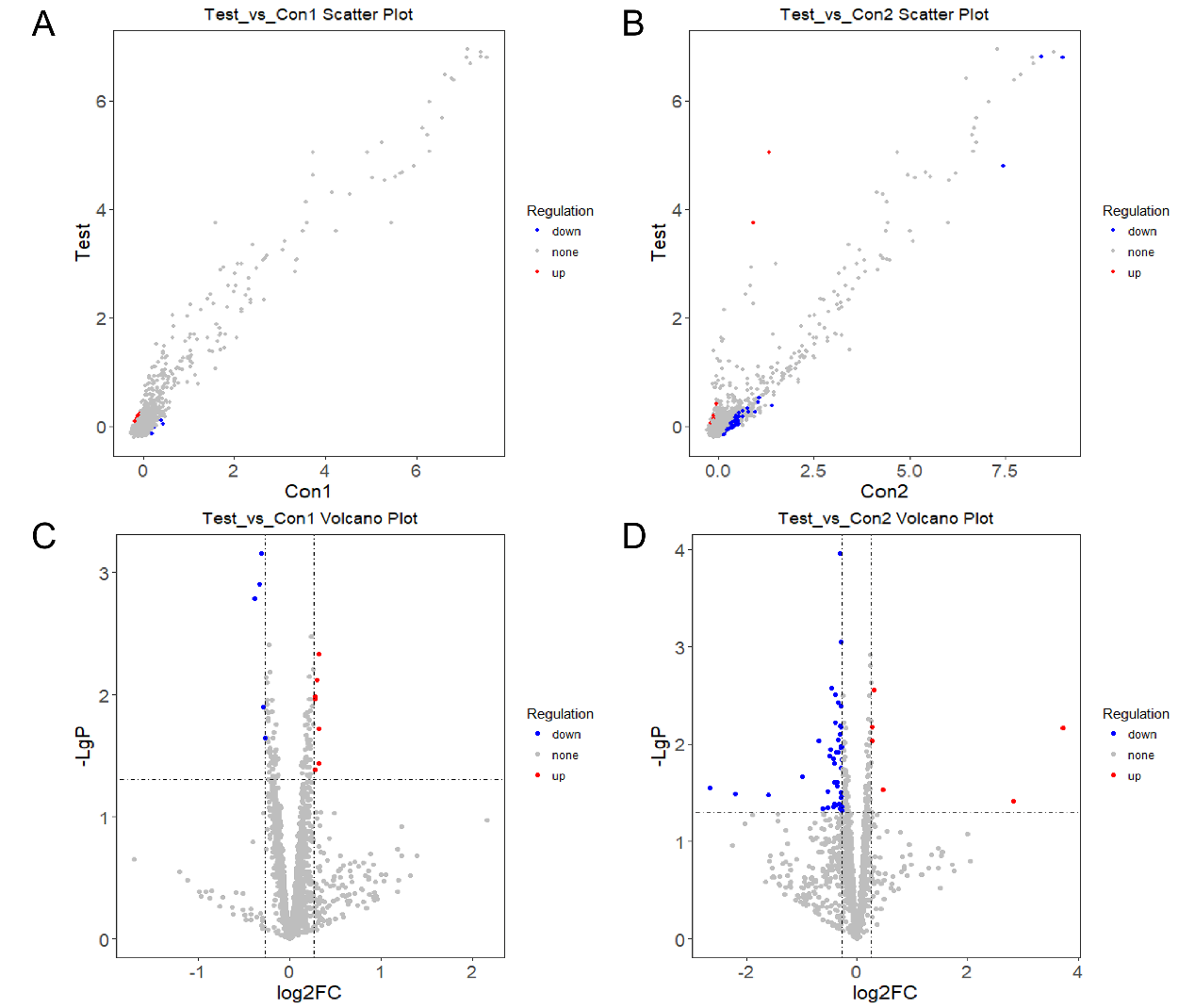
**

**Supplementary Figure S1**. Scatter plots and volcano plots showing the overall distribution of differential expression.

A) and B) scatter plots of differentially expressed microRNAs, where the blue spots mean the down-regulated microRNAs and the red stands the up-regulated microRNAs.

C) and D) volcano plots of differentially expressed microRNAs, where the blue spots mean the down-regulated microRNAs and the red stands the up-regulated microRNAs. Horizontal line represents that P-value equals to 0.05 and the two vertical lines stands that fold change is 1.2 or -1.2.


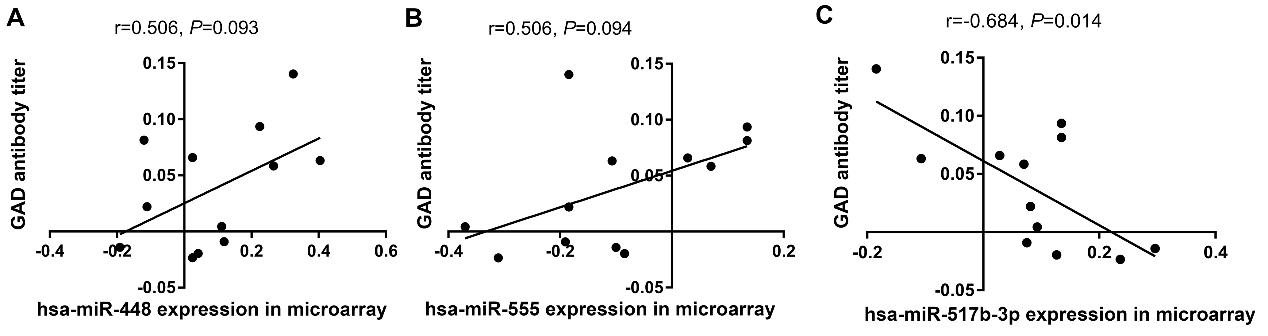


**Supplementary Figure S2**. Correlation between miRNA expression in microarray and GAD antibody titer. The correlations for the miRNAs selected for qRT-PCR validation were analyzed, including (A) hsa-miR-448; (B) hsa-miR-555 and (C) hsa-miR-517b-3p.

**Supplementary Table S1.** The full list of differentially expressed microRNAs in two comparisons.

| **miRNA_ID** | **Group** | **Fold Change** | **P-value** | **Regulation** |
| --- | --- | --- | --- | --- |
| hsa-miR-3175 | Test_vs_Con1 | 1.206842284 | 0.0410234 | up |
| hsa-miR-378c | Test_vs_Con1 | 1.212763508 | 0.0103056 | up |
| hsa-miR-4324 | Test_vs_Con1 | 1.224618861 | 0.00765 | up |
| hsa-miR-4534 | Test_vs_Con1 | 1.248962903 | 0.0362728 | up |
| hsa-miR-585-5p | Test_vs_Con1 | 1.242827485 | 0.0190203 | up |
| hsa-miR-6819-3p | Test_vs_Con1 | 1.245494861 | 0.0046202 | up |
| hsa-miR-1199-3p | Test_vs_Con1 | 1.206518138 | 0.0108952 | up |
| hsa-miR-92a-3p | Test_vs_Con2 | 7.119564048 | 0.03839303 | up |
| hsa-miR-486-5p | Test_vs_Con2 | 13.17642604 | 0.006767935 | up |
| hsa-miR-555 | Test_vs_Con2 | 1.207118258 | 0.006703481 | up |
| hsa-miR-629-3p | Test_vs_Con2 | 1.247146472 | 0.002750923 | up |
| hsa-miR-140-3p | Test_vs_Con2 | 1.378760234 | 0.029500055 | up |
| hsa-miR-4661-5p | Test_vs_Con2 | 1.213634858 | 0.00921371 | up |
| hsa-miR-448 | Test_vs_Con1 | -1.263147729 | 0.0012442 | down |
| hsa-miR-4684-5p | Test_vs_Con1 | -1.203002404 | 0.0224368 | down |
| hsa-miR-1185-1-3p | Test_vs_Con1 | -1.311274904 | 0.001624 | down |
| hsa-miR-889-5p | Test_vs_Con1 | -1.236266895 | 0.0006941 | down |
| hsa-miR-7843-3p | Test_vs_Con1 | -1.221303143 | 0.0125618 | down |
| hsa-miR-517a-3p | Test_vs_Con2 | -1.231612832 | 0.000108927 | down |
| hsa-miR-517b-3p | Test_vs_Con2 | -1.231612832 | 0.000108927 | down |
| hsa-miR-659-3p | Test_vs_Con2 | -1.215996969 | 0.010946894 | down |
| hsa-miR-675-5p | Test_vs_Con2 | -1.238055084 | 0.007974183 | down |
| hsa-miR-371a-5p | Test_vs_Con2 | -1.22604688 | 0.031420061 | down |
| hsa-miR-1290 | Test_vs_Con2 | -1.27034751 | 0.012244812 | down |
| hsa-miR-1249 | Test_vs_Con2 | -1.202616754 | 0.033439315 | down |
| hsa-miR-1292-5p | Test_vs_Con2 | -1.222229752 | 0.035372978 | down |
| hsa-miR-3191-3p | Test_vs_Con2 | -1.215802483 | 0.010402096 | down |
| hsa-miR-4271 | Test_vs_Con2 | -1.307535971 | 0.003108163 | down |
| hsa-miR-3663-3p | Test_vs_Con2 | -1.996548902 | 0.021584774 | down |
| hsa-miR-4428 | Test_vs_Con2 | -1.211257218 | 0.010724971 | down |
| hsa-miR-4481 | Test_vs_Con2 | -1.303522569 | 0.012197191 | down |
| hsa-miR-4516 | Test_vs_Con2 | -6.338894812 | 0.027699002 | down |
| hsa-miR-4525 | Test_vs_Con2 | -1.305543205 | 0.005982167 | down |
| hsa-miR-4526 | Test_vs_Con2 | -1.216123897 | 0.017551609 | down |
| hsa-miR-378i | Test_vs_Con2 | -1.219689183 | 0.004050995 | down |
| hsa-miR-4539 | Test_vs_Con2 | -1.283928938 | 0.026736931 | down |
| hsa-miR-4638-5p | Test_vs_Con2 | -1.20159214 | 0.014454461 | down |
| hsa-miR-4650-3p | Test_vs_Con2 | -1.214901078 | 0.000887191 | down |
| hsa-miR-4690-5p | Test_vs_Con2 | -1.444923212 | 0.044517326 | down |
| hsa-miR-4721 | Test_vs_Con2 | -1.405271678 | 0.013121584 | down |
| hsa-miR-4728-5p | Test_vs_Con2 | -1.62347861 | 0.009139472 | down |
| hsa-miR-4783-3p | Test_vs_Con2 | -1.326606529 | 0.024421878 | down |
| hsa-miR-6089 | Test_vs_Con2 | -4.610257193 | 0.032473906 | down |
| hsa-miR-6090 | Test_vs_Con2 | -3.061922667 | 0.033050899 | down |
| hsa-miR-6132 | Test_vs_Con2 | -1.528801401 | 0.04542581 | down |
| hsa-miR-210-5p | Test_vs_Con2 | -1.217708628 | 0.006634334 | down |
| hsa-miR-504-3p | Test_vs_Con2 | -1.302445008 | 0.041583109 | down |
| hsa-miR-6728-5p | Test_vs_Con2 | -1.333276373 | 0.041066717 | down |
| hsa-miR-6748-5p | Test_vs_Con2 | -1.208410032 | 0.04393349 | down |
| hsa-miR-6796-5p | Test_vs_Con2 | -1.334720506 | 0.015633204 | down |
| hsa-miR-6798-3p | Test_vs_Con2 | -1.272708491 | 0.009031442 | down |
| hsa-miR-6802-3p | Test_vs_Con2 | -1.266068496 | 0.003729785 | down |
| hsa-miR-6806-5p | Test_vs_Con2 | -1.248527903 | 0.041238748 | down |
| hsa-miR-6836-5p | Test_vs_Con2 | -1.207723357 | 0.034268603 | down |
| hsa-miR-6846-5p | Test_vs_Con2 | -1.338337221 | 0.043794296 | down |
| hsa-miR-6865-3p | Test_vs_Con2 | -1.242053852 | 0.006460494 | down |
| hsa-miR-6871-5p | Test_vs_Con2 | -1.393721427 | 0.011497709 | down |
| hsa-miR-6875-3p | Test_vs_Con2 | -1.216354904 | 0.031210429 | down |
| hsa-miR-6879-5p | Test_vs_Con2 | -1.453579619 | 0.030162494 | down |
| hsa-miR-6885-5p | Test_vs_Con2 | -1.373581518 | 0.002666051 | down |
| hsa-miR-6887-5p | Test_vs_Con2 | -1.2819251 | 0.024560681 | down |
| hsa-miR-6893-5p | Test_vs_Con2 | -1.238024572 | 0.04625515 | down |
| hsa-miR-6894-5p | Test_vs_Con2 | -1.295588629 | 0.012127081 | down |
| hsa-miR-7111-5p | Test_vs_Con2 | -1.338118533 | 0.014137218 | down |
| hsa-miR-4433b-5p | Test_vs_Con2 | -1.200427143 | 0.047777493 | down |

**Supplementary Table S2.** The raw CT values from qRT-PCR validation assay.

| miRNA_ID | Sample1 | Sample2 | Sample3 | Sample4 | Sample5 | Sample6 |
| --- | --- | --- | --- | --- | --- | --- |
| CT values for normal (Con1) samples: | | | | | | |
| hsa-miR-448 | 0.0000020000 | 0.0000024110 | 0.0000029000 | 0.0000030000 | 0.0000013110 | 0.0000019000 |
| hsa-miR-555 | 0.0000005300 | 0.0000000300 | 0.0000000820 | 0.0000002300 | 0.0000000700 | 0.0000001020 |
| hsa-miR-517b-3p | 0.0000004790 | 0.0000003980 | 0.0000006000 | 0.0000007190 | 0.0000003980 | 0.0000005600 |
| CT values for type 2 diabetes (Con2) samples: | | | | | | |
| hsa-miR-448 | 0.0000004220 | 0.0000000910 | 0.0000000241 | 0.0000005020 | 0.0000006210 | 0.0000000341 |
| hsa-miR-555 | 0.0000000620 | 0.0000001600 | 0.0000001900 | 0.0000000700 | 0.0000001000 | 0.0000001410 |
| hsa-miR-517b-3p | 0.0000002000 | 0.0000004240 | 0.0000002240 | 0.0000003360 | 0.0000001640 | 0.0000002640 |
| CT values for LADA (Test) samples: | | | | | | |
| hsa-miR-448 | 0.0000008067 | 0.0000004646 | 0.0000009932 | 0.0000006767 | 0.0000007646 | 0.0000004632 |
| hsa-miR-555 | 0.0000003540 | 0.0000003870 | 0.0000005550 | 0.0000006540 | 0.0000002870 | 0.0000001550 |
| hsa-miR-517b-3p | 0.0000000880 | 0.0000001590 | 0.0000000420 | 0.0000001960 | 0.0000001600 | 0.0000001290 |
